# How do healthcare professionals make decisions concerning low-value care practices? Study protocol of a factorial survey experiment on de-implementation

**DOI:** 10.1186/s43058-021-00153-6

**Published:** 2021-05-19

**Authors:** Marta Roczniewska, Ulrica von Thiele Schwarz, Hanna Augustsson, Per Nilsen, Sara Ingvarsson, Henna Hasson

**Affiliations:** 1 Procome Research Group, Medical Management Centre, Department of Learning, Informatics, Management and Ethics, Karolinska Institutet, Stockholm, Sweden; 2 Department of Psychology, SWPS University of Social Sciences and Humanities, Sopot, Poland; 3 School of Health, Care and Social Welfare, Mälardalen University, Västerås, Sweden; 4 Unit for Implementation and Evaluation, Center for Epidemiology and Community Medicine (CES), Stockholm Region, Stockholm, Sweden; 5 Department of Health, Medicine and Caring Sciences, Division of Health and Society, Linköping University, Linköping, Sweden

**Keywords:** Low-value care, De-implementation, De-adoption, Decision-making, Factorial survey experiment, Co-creation

## Abstract

**Background:**

A large number of practices used in health care lack evidence of effectiveness and may be unnecessary or even cause harm. As such, they should be de-implemented. While there are multiple actors involved in de-implementation of such low-value care (LVC) practices, ultimately, the decision to abandon a practice is often made by each health care professional. A recent scoping review identified 6 types of factors affecting the utilization vs. abandonment of LVC practices. These factors concern health care professionals, patients, outer context, inner context, processes, and the characteristics of LVC practice itself. However, it is unclear how professionals weigh these different factors in and how these determinants influence their decisions about abandoning LVC practices. This project aims to investigate how health care professionals account for various factors as they make decisions regarding de-implementation of LVC practices.

**Methods:**

This project will be carried out in two main steps. First, a factorial survey experiment (a vignette study) will be applied to empirically test the relevance of factors previously identified in the literature for health care professionals’ decision-making about de-implementation. Second, interactive workshops with relevant stakeholders will be carried out to develop a framework for professionals’ decision-making and to offer suggestions for interventions to support de-implementation of LVC practices.

**Discussion:**

The project has the potential to contribute to improved understanding of the decision-making involved in de-implementation of LVC practices. We will identify which factors are more important when they make judgments about utilizing versus abandoning LVC practices. The results will provide the basis for recommendations concerning appropriate interventions to support de-implementation decision-making processes.

**Supplementary Information:**

The online version contains supplementary material available at 10.1186/s43058-021-00153-6.

Contributions to the literature

- This project will contribute to de-implementation research by offering valuable insights into health care professionals’ decision-making when they deal with the dilemma of de-implementing low-value practices.
- This study will apply a vignette survey experiment to empirically test which factors influence professionals’ judgments about utilizing vs. abandoning low-value care practices.
- The project will provide theory-based intervention suggestions to support de-implementation of low-value care practices.

## Background

Health care organizations face demands to provide high-quality care and use their resources efficiently. Yet, practices with limited evidence for their benefits keep being prescribed and utilized [1]. The problem is that, on the one hand, it takes a long time to produce research evidence and get it to practical use [2]; on the other, the knowledge quickly becomes outdated and inaccurate when new findings emerge that contradict previous ones [3]. As a consequence, many practices currently used in health care may be unnecessary, ineffective, or even harmful. Hereafter, these non-recommended, ineffective, or harmful practices are referred to as low-value care (LVC). To increase health service efficiency, researchers and practitioners have increasingly turned their interest towards de-implementation [4], i.e., the process of abandoning practices that are not evidence-based [5]. However, the focus has primarily been on *what* should be abandoned, not *how* it should be done. Thus, while lists of non-recommended practices have become common (e.g., Choosing Wisely [6] or the Swedish National Board of Health and Welfare’s “not to do” label in the guidelines), they have little chance of leading to improvements without sufficient knowledge about efficient de-implementation—that is, translating the *what* should be de-implemented into *how* this should actually be carried out [5, 7, 8].

There are multiple actors dealing with LVC and its consequences [9]: national, regional, and organizational health care governance systems [10]; professionals [11]; patients and their associations [12]; and the wider public. Yet, at one point, the ultimate decision to do or not do something is placed in the hands of each health care professional. A survey among physicians demonstrated that they feel great responsibility to make sure their patients avoid unnecessary tests or procedures; overwhelmingly, these physicians also believed that they are in the best position to address the problem, with government agencies ranking far behind [11]. Thus, how and why health care professionals make judgments about using or abandoning a certain practice on the spot in their offices demands attention. Multiple factors influence health care professionals’ decisions. A recent scoping review concerning determinants for the use and de-implementation of LVC in health care identified 6 types of factors concerning: health care professionals, patients, outer context, inner context, process, as well as evidence and LVC practice. Thus, the decision is likely to be influenced by factors across system levels. First, LVC practices have been concerned with healthcare professionals’ individual characteristics and preferences. Particularly, individuals who have invested their time and knowledge in a certain practice may experience a de-implementation process as emotionally demanding. Care practices can be deeply attached to position, status, values, and identity, touching upon issues like what is considered relevant knowledge, as well as ethics and professional roles and expectations. Identified determinants also included professionals’ fear of malpractice. For example, the risk of exposing patients to unnecessary radiation in LVC imaging was perceived to be less significant than the risk of missing a diagnosis [13].

Second, professionals’ use of LVC is also influenced by patient preferences and wishes. In fact, patient requests seem to be a stronger factor for de-implementation compared to implementation of new evidence [14]. For instance, over half of US physicians reported that they would order tests if they were requested by a patient even if they knew these tests were unnecessary [11]. Patient knowledge may act both as a facilitator and a barrier to de-implementation of LVC. When patients are aware of the negative consequences of a specific LVC practice, they are more likely to discuss alternative treatment [15].

Third, professionals’ use of LVC is also determined by factors in the outer context of health care, such as social, political, and geographical factors. For example, the scoping review revealed that in the majority of the studies, having private health insurance was linked to receiving some LVC practices, when compared to other insurance types [14]. Steering mechanisms, including financial aspects, strongly influenced what practices were used, especially when a pay-for-performance system was applied [16]. There is a risk that professionals are encouraged to choose practices based on reimbursement levels rather than the scientific evidence. The review also showed that a policy concerning restricted LVC use was linked to its lower utilization, while advertising about drugs and treatments was identified as influencing more use of LVC.

Fourth, inner context factors such as structural and social environments of hospitals, clinics, and care centers are also determinants of LVC and may influence professionals’ decisions. For example, a lack of care continuity (e.g., having several care providers) was linked to a higher use of LVC practices [14]. Structures in the organization that facilitated using LVC, such as the ownership of equipment used for a particular LVC practice and the ease with which LVC lab tests can be ordered, have also been shown to increase the probability of LVC, along with factors such as reduced support in decision making due to, e.g., absence of teamwork, individual rather than clinic-based decision making, or a lack of feedback/accountability.

Fifth, factors relating to processes of managing LVC may influence decisions about de-implementation, including complexity of a de-implementation process, having routines for managing LVC issues (such as meetings or communication with care recipients [17]), or managerial goals related to lowering LVC. Finally, the characteristics of the scientific evidence undeniably impact how professionals make decisions on whether to provide or abandon a low-value practice. This has to do with how clear the evidence for the low-value practice was and whether an alternative practice is available [18]. Conflicting guidelines and inappropriate or inapplicable guidelines for the patient group also lead to more LVC [14]. Furthermore, physicians’ uncertainty in interpreting research findings and difficulty relating those findings to their own situations contribute to using LVC [19].

In sum, professionals make decisions to de-implement a practice based not only on current evidence but also contingent on patient expectations and characteristics, a wider economic and policy context, prevailing rules, and finally their own knowledge, attitudes, and behaviors. Most prior studies have focused on listing the determinants (i.e., what factors predict provision of LVC) rather than scrutinizing exactly *how* professionals weigh these factors and how these factors impact decisions that either lead to the abandonment or continuation of a LVC practice. Thus, there is lack of knowledge on how professionals carry out decision-making processes concerning providing or abandoning a LVC practice. Focusing on individual decision-making is relevant because health care professionals tend to have considerable autonomy to make decisions as part of their daily work practice. This autonomy may become a source of tension that professionals in health care deal with [20]. On the one hand, the organizations provide highly scripted regulations and goals to professionals, but on the other hand, professionals also need to be responsive to individual cases. They need to navigate this quandary given the boundaries of their professional values and the available organizational resources. In fact, according to the theory of street-level bureaucracy [20], professionals in welfare organizations often lack the necessary resources (e.g., time, information) to provide the highest quality services to each client. Professionals manage this by creating routines and practices and psychologically simplifying both the clients and their environment.

Given the crucial role of health care professionals’ judgments in the de-implementation process, there is a need to test which of the abovementioned factors influence their decision-making related to LVC practices to the highest extent. Overall, one of the main knowledge gaps related to de-implementation deals with understanding professionals’ judgments for solving the potential tension between scientific knowledge and pressure from different actors and aspects of the health care system. Quantifying how factors influence decision-making—both independently and jointly—may offer valuable contribution to practice. Specifically, it may allow development of the best practices for de-implementation processes, identifying barriers, and designing continuing education.

### Aim and research questions

The purpose of this project will be to (1) experimentally test which determinants influence the probability of utilization vs. abandonment of LVC practices most and (2) develop a conceptual framework for professionals’ decision-making about LVC practices to support de-implementation. The following research questions (RQs) will be answered:

1. How do professionals balance and make trade-offs between the distinct determinants, such as their individual characteristics, patient factors, processes as well as health care system inner and outer context, and finally the LVC practice itself?
2. How can professionals’ decision-making to abandon LVC be facilitated at different health care system levels (organizational, regional, and national)?

## Methods

### Overview

This project will be carried out in two main steps. First, a factorial survey experiment (FSE) design [21]—also known as a vignette experiment—will be applied to test how professionals carry out decision-making processes while considering multiple factors that are likely to affect their decisions. This step will be preceded by a pilot study consisting of group interviews where relevant tensions for decision-making will be identified for each group of health care professionals to inform the content of the vignettes.

In the second step, we will develop a conceptual framework for professionals’ decision-making concerning de-implementation and prepare corresponding intervention suggestions. The FSE results, together with the results from relevant literature reviews [14, 22] and group interviews, and a theoretical approach (i.e., theory of street-level bureaucracy) will be used to gradually shift from an intensive focus on how de-implementation decisions are made to the potential solutions to improve the process.

The research will be conducted within primary health care in Sweden. Swedish primary health care is part of the tax-funded health care system and is governed by 21 regions [23]. Several different professional groups work at Swedish primary care centers, which makes it a promising environment to conduct this research. Potential respondents will consist of the following professional groups in primary care: nurses, physicians, physiotherapists, and psychologists. The recruitment of research participants across the two steps will be performed in collaboration with an R&D partner from Stockholm: Academic Primary Care Center (APC). The APC operates in close collaboration with primary care, as well as universities and research institutes. It aims to contribute to quality assurance and development of primary care for staff and students in the Stockholm Region. This collaboration will ensure that the research meets the needs of the primary care and that the project will be well anchored in the organization.

Additional file 1 presents the STROBE checklist [24] of items included in this protocol.

### Study 1

#### Design

A cross-sectional factorial survey experiment will be conducted to investigate the decision-making process of health care professionals concerning potential LVC practices. This approach combines survey research with experimental research [21, 25]. Here, the professionals are asked to make multiple decisions based on random generation of the vignettes (i.e., concrete, fictive clinical case descriptions [21]). An FSE allows analyses of professionals’ judgments and is particularly suitable for investigating complex situations with multiple stimuli. This approach has the unique ability to simultaneously measure the independent effects of respondents and situational factors without the need to give participants every possible combination of variables. This method is widely used to study health care professionals’ decision-making, e.g., [26–29]. FSE is a suitable method to study the often, and somewhat, sensitive issue of LVC because it provides a less personal and therefore less threatening way of exploring the topic [21]. Furthermore, professionals’ decision-making is often challenging to study with conventional social science methods such as interviews and observations [21]. Although the decisions made in an FSE are hypothetical and do not replicate real life, it is considered suitable for providing an in-depth insight into decision-making processes, which this project aims to do. Apart from manipulating the factors in the vignettes, we will collect relevant data about health care professionals, which may affect how they make their decisions. Following the previous literature [14, 30], we will consider their length of clinical experience, fear of possible litigation, and a tendency to worry that they may miss a certain diagnosis. For the sensitivity analysis, we will control gender.

#### Study materials—vignettes

Based on a recent scoping review summarizing factors affecting de-implementation [14], we will identify potential tensions in decision-making processes among health care professionals. Tensions consist of situational attributes that together constitute a potential dilemma for a professional. For instance, a tension can be a situation where a low-value practice is strongly requested by a patient and there are low costs related to providing it, but the evidence is clear that it has no clinical benefits. The factors identified in the literature will be discussed with the R&D partner to validate the ecological validity of the material. Thereafter, we will create drafts for vignettes, i.e., descriptions that comprise a series of sentences in a fixed order containing the relevant dimensions for a certain decision (see Table 1 for examples of dimensions and their values).

**Table 1:** Vignette dimension examples and their values

Dimension example	Variable values
Patient expectation	1. Strongly demands practice
	2. Neutral about practice
	3. Strongly against practice
Patient’s age	1. Young adult
	2. Middle-aged
	3. Elderly
Cost of the practice	1. Low
	2. High
Trust towards the source of the guideline identifying practice as LVC	1. Yes
	2. No
Evidence for the lack of benefits of the LVC practice	1. Clear
	2. Unclear

**Fig. 1:**
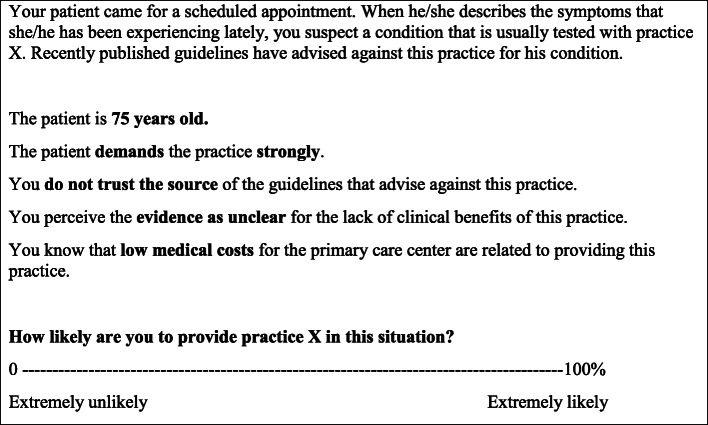
Randomly populated vignette example

The level or presence of a certain dimension (e.g., patient wishes for a certain practice) is randomly varied among the vignettes and respondents (see Fig. 1). These vignettes present a story that is relevant for a specific profession, although the tensions included can be similar among professional groups (i.e., patient requests and costs in relation to evidence).

Following the recommendations for FSEs, we will use approximately 7 ± 2 dimensions with 2–3 levels for each of these [21, 25]. In this way, we will be able to empirically test how differences in the dimensions impact the participants’ decisions (e.g., a low-value practice is strongly OR not at all required by a patient, low OR medium OR high costs are related to providing it, and the evidence is clear OR unclear for the lack of clinical benefits). Judging several similar, but not identical, situations by each respondent allows the FSE to decompose the structure of the individuals’ answer behaviors and thereby uncover the impact of the different factors [21]. The dependent variable will be the respondent’s decision to provide the practice described in the vignette, using a visual analog scale from 0% (extremely unlikely) to 100 % (extremely likely).

The vignette universe will be generated by crossing all of the possible combinations (Cartesian product) of the vignette dimensions’ categories to ensure orthogonality across the factors [21]. The experimental design (i.e., vignette universe) will be divided into different decks (blocks) presented to different respondents. This way, we will be able to use a larger overall number of vignettes to enhance statistical efficiency [21]. For blocking to decks, we will use deliberate blocking techniques based on design efficiency (D-efficiency) [21]. A minimum of five respondents will assess each deck [21]. We will use the SAS macro “%mktblock” to ensure a randomized distribution of the whole vignette universe over the decks in order to attain maximum statistical efficiency. By distributing the different questionnaires as evenly as possible, we will ensure that the correlations between dimensions are close to 0 and not significant.

The drafts for vignettes will be pilot-tested and further developed in the group interviews with a selection of professionals from the participating primary care centers [31]. Thus, the clinical relevance of the tensions and factors will be further tested. This is a crucial step as the strength of the methodology used in the next project step relies on how well the respondents identify with the situations described in the vignettes [21]. Use of professionals’ expertise is a common way to ensure that the vignettes represent the realities these are portraying and, in this way, increase the validity of the methodology [21]. We will use a purposeful sample that varies in terms of gender and length of profession. This diversity seeks to increase the relevance of the vignettes. The group interviews will start with a presentation of the vignette drafts, which thereafter will be discussed by the participants. The moderator will pose questions about the formulation of the vignettes, their relevance, and other potential factors that might impact decision-making. The group interviews will continue until saturation is achieved within each professional group. We estimate that approximately 2–3 group discussions for each professional group are needed, for a total of 8–12 group interviews. To finalize the vignettes, a smaller number of professionals will be consulted for a cognitive pretest based on think-aloud techniques [28]. They will be asked to read the vignettes with the different dimensions and levels to check whether these are easy to read and understand.

#### Participants and procedure

First, managers for each primary care center will be approached to anchor the project and to obtain approval for the professionals’ participation. Based on our previous experiences conducting surveys with an organization, we estimate that at least 60% of the centers will participate (i.e., at least 42 centers). The number of professionals at each center varies, commonly from 15 to 20. This estimate would give us a population of professionals that is large enough for the planned survey (at least 630–840 individuals, estimated response rate 60%) [21], although a more thorough power calculation will be performed when the vignettes have been finalized, i.e., the number of factors and dimensions is determined. SAS software will be used for the power analysis. We plan to use quota sampling as our sampling technique and multiple regression analyses for the data analysis. Although simple randomization is common, the current FSE literature proposes that quota sampling has advantages in understanding confounded parameters. We will, therefore, based on the results of the literature review, investigate whether a specific characteristic in the respondent population (e.g., gender) can be an appropriate factor for stratification in a quota sampling. Otherwise, we will use random sampling and carefully target the potential limitations of this method.

Each survey respondent will answer 9–12 vignettes. This number was determined based on prior research showing that health care professionals are able to provide reliable answers to this number of vignettes [21, 26, 29]. The number of vignettes will be based on the time the participant are estimated to be able to spend on the survey (max. 15 minutes), the difficulty and complexity of the vignettes and statistical considerations (e.g., power) [21].

#### Statistical analysis

Because each respondent will evaluate multiple vignettes, this implies a hierarchical data structure in which the responses (decisions to provide the LVC practice in each scenario) are nested within respondents (healthcare professionals). To address this violation of the classical regression assumption of uncorrelated error terms, mixed-effects models will be performed with vignettes’ dimensions as level 1 and respondents as level 2. First, a random intercept-only model with no predictors (i.e., null model) will be performed to calculate an intraclass correlation coefficient (ICC) and to benchmark model fit. Then, vignette dimension variables and respondent variables, as well as their interactions, will be added sequentially. In the case of low ICC coefficients, regular multivariable regressions will be performed alongside multilevel analyses for a sensitivity test.

### Study 2

#### Participants and procedure

The knowledge gained about how professionals make decisions when dealing with LVC practices will be utilized for creating practical suggestions that can be valuable for key actors involved in health care organizations and steering the de-implementation of LVC. Specifically, we will develop a conceptual framework for professionals’ decision-making concerning de-implementation based on the results from the FSE (Study 1), relevant literature reviews [14, 22] and group interviews, and a theoretical approach (i.e., theory of street-level bureaucracy). These sources will be used to gradually shift from an intensive focus on how de-implementation decisions are made to the potential solutions to improve the process. It will be iteratively tested and further developed with the professionals, the local R&D partner, and other researchers in scientific conferences in order to obtain both optimal scientific rigor and practical usefulness.

Based on this framework, intervention suggestions (e.g., checklists and decision support tools) for facilitating professionals’ decision-making concerning the de-implementation of low-value practices will be developed. Suggestions will be relevant for all levels in the health care system (e.g., national, regional, and organizational) and will focus on facilitating professionals’ decision to engage in de-implementation. The intervention suggestions will be developed in workshops with professionals and other stakeholders involved in organizations for de-implementing LVC.

The conceptual framework will be presented to guide participants’ efforts to propose suitable interventions. We will use an interactive, 2-step process [32] in which participants are first asked to brainstorm potential interventions by answering a specific question (e.g., What can facilitate professionals’ decision-making about the provision of LVC?). Thereafter, these suggestions are rated by the participants for their potential impact, feasibility as well as strengths and weaknesses in health care. Based on previous experience with this method [33], approximately 3–6 workshops will be conducted.

## Discussion

This project aims to contribute to knowledge and practice concerning de-implementation of LVC practices by focusing on the decision-making processes among health care professionals. More specifically, the study will provide three main contributions to the topic. First, the project’s novel contribution lies in approaching the well-known research-to-practice gap by investigating how decisions about abandoning the LVC practices are made rather than by studying which practices need to be abandoned. Thus, the project targets one of the main knowledge gaps related to de-implementation: understanding health care professionals’ perspective and their judgments for solving potential tensions related to the provision of LVC practices. A survey among physicians demonstrated that a majority of them feel that a great deal of responsibility falls on them to make sure their patients avoid unnecessary tests or procedures; these physicians also believed that they are in the best position to address the problem [11]. By taking this perspective, this project will contribute to research and practice by investigating how healthcare professionals make their decisions to prescribe or to avoid a LVC test or procedure. Specifically, we will provide understanding on the decision-making processes by observing how healthcare professionals balance factors identified as determinants for the use of LVC [14]. By juxtaposing and combining these factors, we may be able to identify which of them plays a more deciding role in their judgments, but also which combinations of factors lead to a more frequent decision not to utilize a practice considered as low value.

Second, the project will develop a conceptual framework for professionals’ decision-making about LVC practices. This can be used as a framework in future studies that aim to answer which factors are relevant for health care professionals’ decisions regarding a specific LVC practice. We have an opportunity to compare the proposed framework with existing frameworks for implementation (i.e., putting new practices in place) as it is possible that the decision-making processes differ when something new is implemented as compared to when something is de-implemented. Likely, there will be some overlap between implementation and de-implementation with regard to the determinants, but their relative importance may differ between implementation and de-implementation [22]. Thus, this step will highlight potential differences in these two processes and contribute to specifying the distinctiveness of the de-implementation as a separate research and practice area [34].

Third, evidence-based intervention suggestions will be developed to offer practical solutions to health care professionals and managers. These interventions will aim at facilitating professionals’ decision-making concerning the de-implementation of low-value practices. The suggestions can be suitable for all levels in the health care system (e.g., national, regional, and organizational) depending on their potential impact on facilitating professionals’ de-implementation decision-making. Because decision-making about de-implementation is an under-researched area, not many solutions are available to facilitate this process for health care professionals. This project will offer the research community the opportunity to evaluate the impact of these solutions on de-implementation processes in future studies. These suggestions can later be evaluated for their impact in different clinical settings. These workshops are also an important step in disseminating the findings and providing knowledge on de-implementation to health care stakeholders. Effective interventions for decreasing the use of ineffective practices in health care will lead to improved quality of care provided in the future.

## Supplementary Information

**Additional file 1.** The STROBE checklist.

## Data Availability

The datasets used will be available from the corresponding author on reasonable request.

## Abbreviations

LVC: Low-value care
FSE: Factorial survey experiment
